# New eight genes identified at the clinical multidrug-resistant *Acinetobacter baumannii* DMS06669 strain in a Vietnam hospital

**DOI:** 10.1186/s12941-017-0250-9

**Published:** 2017-11-14

**Authors:** Nguyen Si-Tuan, Hua My Ngoc, Pham Thi Thu Hang, Cuong Nguyen, Pham Hung Van, Nguyen Thuy Huong

**Affiliations:** 1 Department of Biotechnology, Faculty of Chemical Engineering, Ho Chi Minh City University of Technology, HCM National University, Ho Chi Minh City, Vietnam; 2 Molecular Medicine Laboratory, Faculty of Medical Microbiology, Thong Nhat Dong Nai General Hospital, Bien Hoa City - Dong Nai Province, Vietnam; 3 Department of Microbiology, Faculty of Biology, Ho Chi Minh City University of Natural Science, HCM National University, Ho Chi Minh City, Vietnam; 4 Department of Bioinformatics and Medical Statistics, Vinmec Research Institute of Stem Cell and Gene Technology, Hanoi, Vietnam; 5 The HCM Society of Clinical Microbiologists, Ho Chi Minh City, Vietnam; 6 Faculty of Medical Biochemistry, Thong Nhat Dong Nai General Hospital, Bien Hoa City - Dong Nai Province, Vietnam

**Keywords:** *Acinetobacter baumannii*, Multidrug resistance, Draft genome assembly, Next generation sequencing

## Abstract

**Background:**

*Acinetobacter baumannii* is an important nosocomial pathogen that can develop multidrug resistance. In this study, we characterized the genome of the *A. baumannii* strain DMS06669 (isolated from the sputum of a male patient with hospital-acquired pneumonia) and focused on identification of genes relevant to antibiotic resistance.

**Methods:**

Whole genome analysis of *A. baumannii* DMS06669 from hospital-acquired pneumonia patients included de novo assembly; gene prediction; functional annotation to public databases; phylogenetics tree construction and antibiotics genes identification.

**Results:**

After sequencing the *A. baumannii* DMS06669 genome and performing quality control, de novo genome assembly was carried out, producing 24 scaffolds. Public databases were used for gene prediction and functional annotation to construct a phylogenetic tree of the DMS06669 strain with 21 other *A. baumannii* strains. A total of 18 possible antibiotic resistance genes, conferring resistance to eight distinct classes of antibiotics, were identified. Eight of these genes have not previously been reported to occur in *A. baumannii*.

**Conclusions:**

Our results provide important information regarding mechanisms that may contribute to antibiotic resistance in the DMS06669 strain, and have implications for treatment of patients infected with *A. baumannii.*

**Electronic supplementary material:**

The online version of this article (10.1186/s12941-017-0250-9) contains supplementary material, which is available to authorized users.

## Background

The nosocomial pathogen *Acinetobacter baumannii* has become a serious world-wide health concern, as it has in recent decades acquired resistance to an array of antibiotics [1–3]. The development of resistance to carbapenem is of particular concern [4, 5]. *A. baumannii* is responsible for 2–10% of all Gram-negative bacterial infections in intensive care units, and causes significant mortality in infected patients [6, 7]. Infection with multidrug resistant (MDR) *Acinetobacter* strains is associated both with elevated mortality rates and longer hospital treatment times [8].

Recent advances in next-generation sequencing have facilitated the rapid characterization of whole-genome sequences from microbial species [9]. In this study, we used the Illumina HiSeq® platform to carry out de novo assembly of the genome of the *A. baumannii* strain DMS06669 to identify genes related to antibiotic resistance. DNA samples were obtained from the sputum of a male patient with hospital-acquired pneumonia. Gene prediction and functional annotation were carried out using public databases, followed by construction of a phylogenetic tree and whole-genome comparison. Antibiotic resistance of DMS06669 to multiple antibiotics was characterized using an antibiotic susceptibility assay test, and potential antibiotic resistance genes in DMS06669 were predicted from functional annotation analysis. Identification of these genes is an important step in elucidating mechanisms underlying antibiotic resistance in *A. baumannii*.

## Methods

### Isolation and identification of *A. baumannii* strain DMS06669

The *A. baumannii* strain DMS06669 was isolated from a sputum specimen obtained from a 49-year-old male patient with hospital-acquired pneumonia at Thong Nhat Dong Nai General Hospital in Dong Nai, Vietnam. This isolate was cultured at 37 °C on blood agar and MacConkey agar. The presence of the blaOXA-51 gene was used to confirm that the isolate was a strain of *A. baumannii* [10].

### Antibiotic susceptibility assay test

Microbiological isolation and identification of the *A. baumannii* strain DMS06669 was performed using classic methods and verified by the Phoenix system (BD Diagnostics, Sparks, MD, USA). Antimicrobial susceptibility testing was performed using the minimum inhibitory concentration (MIC) breakpoint method according to Clinical and Laboratory Standards Institute (CLSI) guidelines. Antimicrobials tested included amikacin, aztreonam, ampicillin/sulbactam, ciprofloxacin, ceftazidime, ceftriaxone, colistin, cefepime, cefoxitin, ceftazolin, gentamicin, imipenem, meropenem, levofloxacin, trimethoprim/sulfamethoxazole, ticarcillin/clavulanic acid, tigecycline, and piperacillin/tazobactam.

### DNA preparation and sequencing

Genomic DNA of *A. baumannii* DMS06669 was extracted using the Wizard DNA extraction kit (Promega, USA), and then sequenced (1st BASE, Singapore) on an Illumina HiSeq 2000, resulting in 150 base pair (bp) paired-end reads and an average coverage of 120-fold.

### Data preprocessing and de novo genome assembly

Raw paired-end reads were assessed and quality control was carried out using FastQC (http://www.bioinformatics.babraham.ac.uk/projects/fastqc/) and Trimmomatic [11] (parameters: ILLUMINACLIP:2:30:10 LEADING:3 TRAILING:3 SLIDINGWINDOW:10:30 MINLEN:100) to obtain a set of clean paired-end reads. After preprocessing, FastQC was again used to report features of the preprocessing libraries and to verify the effectiveness of read trimming. After filtering, short reads were assembled using the SPAdes Genome Assembler [12] and contigs longer than 300 bp were retained. Then, those contigs were launched in a multi-draft based analysis to reorder through MeDuSa software [13], using *A. baumannii* ATCC 17978 as a reference.

### Genome annotation

Prodigal (version 2.6.2) [14] was used for gene prediction in the *A. baumannii* DMS06669 draft genome, while tRNAscan-SE [15] and RNAmmer [16] were used for tRNA and rRNA (5S, 16S, and 23S) identification. Tandem Repeat Finder (http://tandem.bu.edu/trf/trf.html) [17] and CRISPR Finder (http://crispr.u-psud.fr/Server/) [18] were used to find tandem repeat sequences and clustered regularly interspaced short palindromic repeats (CRISPRs). Insertion sequences (ISs) were identified using ISfinder (https://www-is.biotoul.fr//). For functional classification of the predicted genes, BLASTp [19] was used to align amino acids of predicted genes against the Clusters of Orthologous Groups (COG) database [20] with an expected value threshold of 10e^−3^ using the Conserved Domains Database (CDD) batch server [21] (https://www.ncbi.nlm.nih.gov/Structure/bwrpsb/bwrpsb.cgi). Amino acid sequences were aligned using default parameters, and the description of the best hit (highest alignment length percentage and match identity) was assigned as the predicted gene. All annotated genes were then categorized based on their COG classification. ResFinder (version 2.1; https://cge.cbs.dtu.dk/services/ResFinder/) was used to identify potential antibiotic resistance genes, using recommended similarity thresholds [22].

### Construction of phylogenetic tree

16S rRNA sequences were extracted from the draft genome of DMS06669, and 16S rRNA sequences of 21 *A. baumannii* strains (including 1656-2, AB0057, AB030, AB031, AB307-0294, AC29, ACICU, ATCC17978, AYE, AbH12O-A2, BJAB07104, BJAB0715, BJAB0868, D1279779, LAC-4, MDR-TJ, MDR-ZJ06, SDF, TCDC-AB0715, TYTH-1, and ZW85-1) were downloaded using the Kyoto Encyclopedia of Genes and Genomes (KEGG) database [23]. Evolutionary analyses of 16S rRNA sequences were carried out using ClustalW (version 2.1) to multiple aligned sequences. The maximum-likelihood phylogenetic tree was then constructed using PHYLIP (version 3.695) [24] with the bootstrap algorithm set to 500, and the phylogenetic tree was visualized using FigTree (version 1.4.3) (http://tree.bio.ed.ac.uk/software/fgtree/).

## Results and discussion

### Antibiotic resistance of A. *baumannii* DMS06669

The antibiotic susceptibility profile for the DMS06669 strain is shown in Table 1. The *A. baumannii* DMS06669 strain was resistant to all antibiotics tested with high level of MIC values except colistin and tigecycline.

**Table 1:** Susceptibility profile of *A. baumannii* strain DMS06669

Antibiotic drug	MIC (µg/ml)
Ampicillin/sulbactam	32/16
Ceftazidime	32
Ciprofloxacin	4
Levofloxacin	4
Imipenem	16
Meropenem	16
Gentamicin	16
Colistin	1
Amikacin	64
Piperacillin/tazobactam	128/8
Ticarcillin/clavulanic acid	128/4
Cefepime	32
Cefoxitin	32
Cefazolin	16
Ceftriaxone	8
Tigecycline	1
Aztreonam	32
Trimethoprim/sulfamethoxazole	8/76

### Assembly and annotation of the genomic sequence

Raw reads from the Illumina HiSeq mahcine were assessed with FastQC and filtered using Trimmomatic to produce a clean set of reads. These whole genome DNA samples yielded 4,750,865 raw paired-end reads with lengths of 150 bp. After removal of adapters and low-quality data (quality scores ≤ 30), 3,768,594 clean reads (79.3% of the initial total) remained (Table 2).

**Table 2:** Results of genome assembly and annotation of *A. baumannii* strain DMS06669

Feature	Statistics
Pair-end raw reads	4,750,865
Pair-end clean reads (remaining percentage)	3,768,594 (79.32%)
Length of total draft genome length (bp)	4,369,281
Scaffolds	24
Length of scaffold (N50)	4,207,939
GC content (%)	38.9
Coding sequences	4101
tRNAs	63
rRNAs	3
CRISPR sequences	2^a^
Tandem repeat sequences	6
Insertion sequences	62

^a^Includes one questionable (non-confirmed) sequence

The clean paired-end read dataset was used for de novo genome assembly using the SPAdes Genome Assembler and reordering by MeDuSa, and resulted in 24 scaffolds, with a total draft genome length of 4,369,281 bp (N50 of 4,207,939 bp and GC content of 38.9%). From genome annotation analysis, we detected 4101 coding sequences, 63 tRNA sequences, 3 rRNA sequences, 2 CRISPR sequences, 6 tandem repeat sequences, and 62 predicted ISs (Table 2).

### Functional annotation of the genomic sequence of *A*. *baumannii* strain DMS06669

**Fig. 1:**
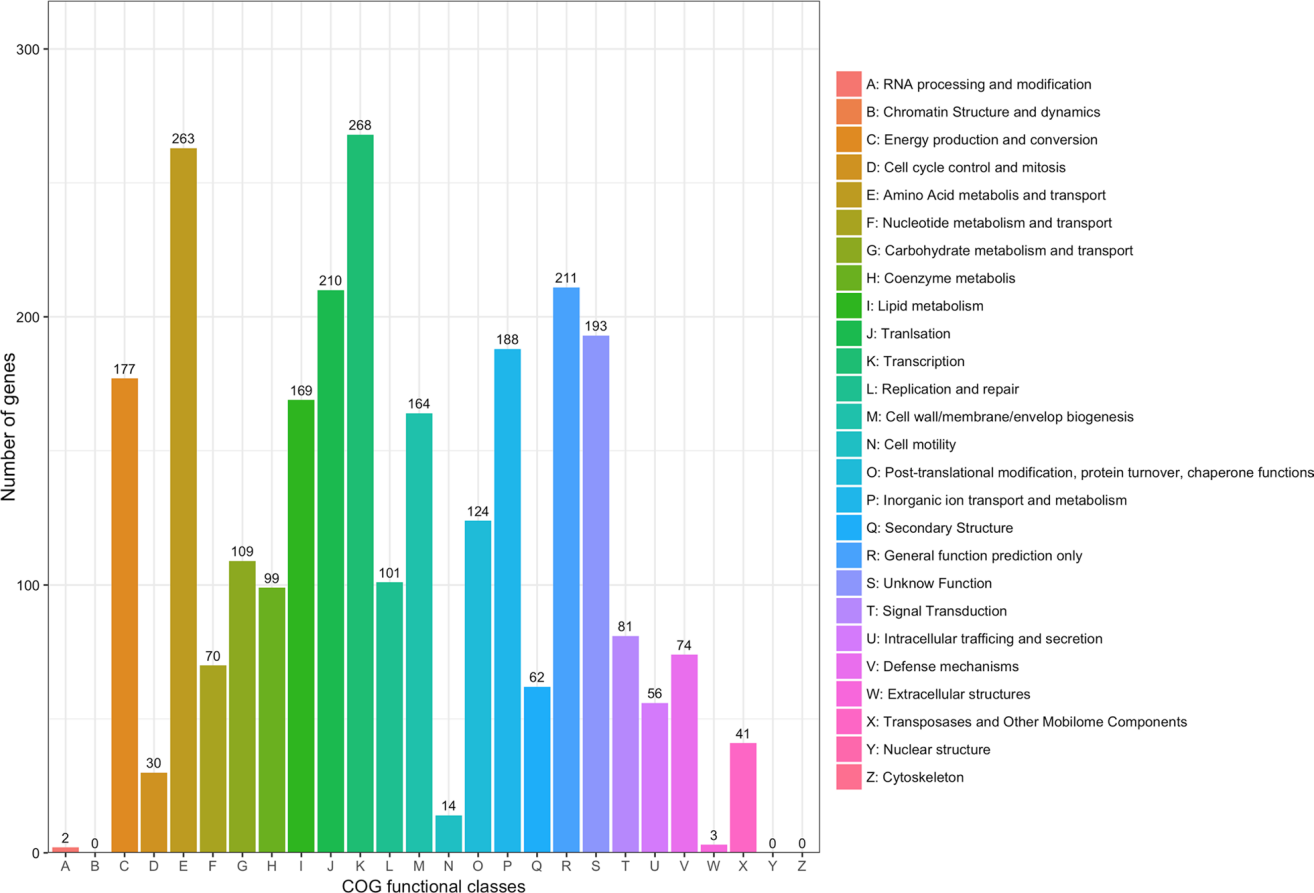
COG functional classification of coding sequences. A total of 3066 coding sequences were annotated and assigned to 25 functional categories

The COG database phylogenetically classifies proteins encoded by completely sequenced genomes. Of the 4101 coding sequences identified in the DMS06669 genome, 3066 sequences (74.8%) were annotated and classified into 25 functional categories (Fig. 1). The identity ratio in this study was similar to those obtained in studies of *A. baumannii* strains MDR-TJ, MDR-SHH02, and XH386 (ST208) [22, 25, 26]. Among the aligned COG classifications, the “transcription” category comprised the largest group (268 coding sequences, 8.7% of the total number), followed by “amino acid transport and metabolism” (263 coding sequences, 8.6%), “general function prediction only” (211 coding sequences, 6.9%), and “translation” (210 coding sequences, 6.8%). In addition, 193 coding sequences were classified as having “unknown function”. No coding sequences were assigned to functional categories of “chromatin structure and dynamics”, “nuclear structure”, or “cytoskeleton” (Fig. 1).

**Fig. 2:**
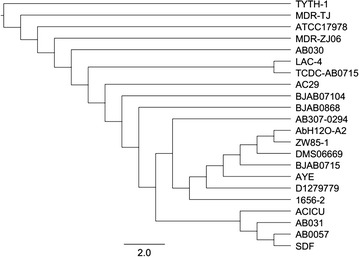
16S rRNA phylogenetic analysis showing the evolutionary relationship between *A. baumannii* strains. The tree was generated with PHYLIP (version 3.695) with the bootstrap algorithm set to 500 and standard settings. The scale bar represents the branch lengths

Based on genomes from published *A. baumannii* strains on the KEGG database, we performed phylogenetic analysis of 16S rRNA to identify genetic relatedness of strains. Figure 2 shows that DMS06669 is most closely related to the ZW-85 and AbH120-A2 strains.

### Identification of antibiotic resistance genes

To identify *A. baumannii* DMS06669 genes related to antibiotic resistance, coding sequences were submitted to ResFinder [22]. Table 3 lists genes that may contribute to *A. baumannii* DMS06669 resistance to aminoglycosides, beta-lactams, macrolides, lincosamide streptogramin B, phenicols, rifampicin, sulphonamides, tetracyclines, and trimethoprim.

**Table 3:** Resistance-associated genes identified in *A. baumannii* strain DMS06669

Predicted gene	Resistance gene	Antibiotic class	% identity	HSP length/query length
DMS06669_scf_4_1	*aadA16*	Aminoglycoside resistance	99.7	846/846
DMS06669_scf_2_1	*aadB*	Aminoglycoside resistance	100.00	534/534
DMS06669_scf_23_3	*aadA1*	Aminoglycoside resistance	99.9	792/792
DMS06669_scf_22_2	*rmtB*	Aminoglycoside resistance	100.00	756/756
DMS06669_scf_2_2	*blaVEB*-*7*	Beta-lactam resistance	99.9	900/900
DMS06669_scf_23_2	*blaOXA*-*10*	Beta-lactam resistance	100.00	801/801
DMS06669_scf_18_1	*blaOXA*-*58*	Beta-lactam resistance	100.00	843/843
DMS06669_scf_1_2828	*blaADC*-*25*	Beta-lactam resistance	96.3	1152/1152
DMS06669_scf_11_9	*blaNDM*-*1*	Beta-lactam resistance	100.00	813/813
DMS06669_scf_1_1731	*blaOXA*-*64*	Beta-lactam resistance	100.00	825/825
DMS06669_scf_23_1	*cmlA1*	Phenicol resistance	99.1	1260/1260
DMS06669_scf_21_2	*floR*	Phenicol resistance	98.3	1214/1215
DMS06669_scf_5_1	*sul1*	Sulphonamide resistance	100.00	840/840
DMS06669_scf_8_3	*tet(39)*	Tetracycline resistance	99.9	1122/1122
DMS06669_scf_13_10	*mph(E)*	Macrolide resistance	100.00	885/885
DMS06669_scf_13_11	*msr(E)*	Macrolide, lincosamide, and streptogramin B resistance	100.00	1476/1476
DMS06669_scf_16_1	*ARR*-*3*	Rifampicin resistance	100.00	453/453
DMS06669_scf_4_2	*dfrA27*	Trimethoprim resistance	100.00	474/474

**Fig. 3:**
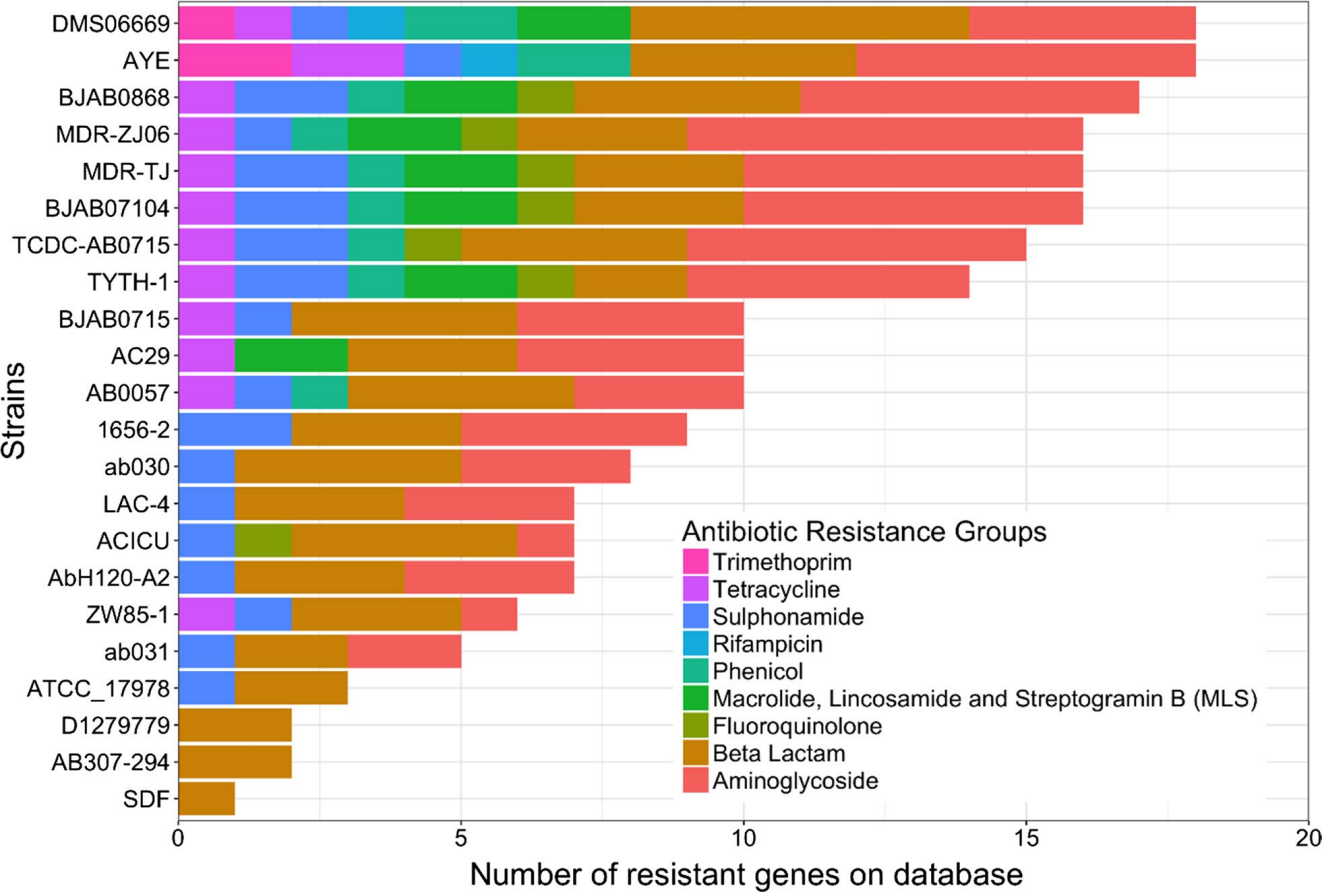
Antibiotic resistance in *A. baumannii* strain DMS06669 and 21 other *A. baumannii* strains

Among 22 *A. baumannii* strains, the DMS06669 strain had resistance to the highest number of types of antibiotics (including eightHo Chi Minh City of nine antibiotic classes, with susceptibility only to fluoroquinolones; Fig. 3). DMS06669 was followed by AYE, BJAB0868, MDR-ZJ06, MDR-TJ, and BJAB07104 strains, all of which have been reported to be MDR strains. The absence of resistance to fluoroquinolones in the DMS06669 strain was consistent with MIC analysis (Table 1) when the antibiotic susceptibility profile shows that MIC values of ciprofloxacin and levofloxacin was at intermediate level (4 µg/ml for each antibiotic). Detailed results of all antibiotic resistance genes in each class of antibiotics are included in Additional file 1: Table S1.

Genes related to gentamicin (*aadB* [27]) and amikacin (*rmtB* [28]) resistance were found in the DMS06669 strain. MIC values for DMS06669 susceptibility to gentamicin and amikacin were high, measured at 16 and 64 µg/ml, respectively (Table 1). The *rmtB* aminoglycoside resistance gene has not previously been reported to occur in *A. baumannii*. Additionally, we found the *aadA1* gene [29], involved in streptomycin resistance, and the *aadA16* gene (not reported in *A. baumannii* before) [30], involved in spectinomycin resistance, in the DMS06669 genome.

Two genes related to macrolide, lincosamide, and streptogramin B resistance were found in the DMS06669 strain: *mphE* [31], associated with resistance to erythromycin (a macrolide antibiotic), and *msrE* [32], associated with streptogramin resistance. The presence of these genes in other MDR strains, including BJAB0868, BJAB07104, TYTH-1, MDR-ZJ06, AC29, and MDR-TJ, has previously been reported (Additional file 1: Table S1).

We found genes associated with resistance to phenicol, rifapicin, and sulphonamide antibiotics, including two genes already known to occur in *A. baumannii*: *cmlA1*, associated with chloramphenicol resistance [33], and *sul1*, associated with sulfamethoxazole resistance [34]. We identified two additional genes in this category that occur only in the DMS06669 strain: *floR*, associated with chloramphenicol and florfenicol resistance [35]; and *arr*-*3,* associated with rifampin (also known as rifampicin), rifaximin, rifabutin, and rifapentine antibiotic resistant groups [36].

Two predicted DMS06669 genes were similar to the *tet(39)* and *dfrA27* genes, which are associated with resistance to tetracycline and trimethoprim, respectively. The presence of homologs to these genes in *A. baumannii* has not previously been reported. The *tet(39)* gene has been reported to confer resistance to tetracycline [37], which has a structure similar to that of tigecycline [38]. However, according to our analysis (Table 1), DMS06669 was weakly resistant to tigecycline (MIC value of 1 µg/ml), suggesting that *tet(39)* is not associated with tigecycline resistance. It has been reported that the *dfrA27* gene confers trimethoprim resistance, and the complex of *dfrA27* and *aadA16* genes have been reported in the MDR *E. coli* strain 1387 [30]. We found that this complex was also present in the DMS06669 strain.

Interestingly, DMS06669 contained six genes associated with resistance to beta-lactamase. The *blaVEB*-*7* and *blaADC*-*25* genes have been associated with resistance to cephalosporins (a group that includes cefepime, cefoxitin, cefazolin, and ceftriaxone) and aztreonam [39, 40], in agreement with MIC analysis (Table 1). DMS06669 also contained the genes *blaOXA*-*10* [41], *blaOXA*-*58* [42], *blaOXA*-*64* [43], and *blaNDM*-*1* [44], which are thought to confer resistance to carbapenems (a group that includes meropenem and imipenem). Among these genes, *blaOXA*-*64* and *blaVEB*-*7* genes have not previously been reported to occur in *A. baumannii* strains. Moreover, both *blaNDM*-*1* and *blaOXA*-*58* genes that are found in the DMS06669 strain have not previously been reported to occur in the same strain.

## Conclusions

In this study, the genome of the *A. baumannii* strain DMS06669 was sequenced and de novo genome assembly was carried out, yielding 24 scaffolds. Phylogeny analysis was conducted from 16S rRNA extracted from the assembled genome. A total of 4101 coding sequences were predicted and annotated, leading to identification of 18 predicted genes associated with resistance to eight classes of antibiotics. Eight of these genes have not previously been reported to occur in *A. baumannii*. Furthermore, there were two antibiotic resistance genes (*blaNDM*-*1* and *blaOXA*-*58* genes) found in the DMS06669 strain that have not been reported together in any *A. baumannii* strain. Our analysis significantly expands understanding of the *A. baumannii* genome, and represents an important step in elucidating the molecular mechanisms of antibiotic resistance in this species.

## Additional file

**Additional file 1: Table S1.** Detailed result of all antibiotic resistance genes in each antibiotic class of *A. baumannii* DMS06669 and other 21 *A. baumannii* strains (downloaded from KEGG database) from searching ResFinder database (genes with name in red color were not reported in other *A. baumanni* strains before).

## Abbreviations

MDR: multidrug resistant
MIC: minimum inhibitory concentration
CLSI: Clinical and Laboratory Standards Institute
bp: base pair
CRISPRs: clustered regularly interspaced short palindromic repeats
IS: insertion sequences
COG: Clusters of Orthologous Groups
CDD: conserved domains database
KEGG: Kyoto Encyclopedia of Genes and Genomes

## References

[1] Poirel L, Nordmann P (2006). Carbapenem resistance in *Acinetobacter baumannii*: mechanisms and epidemiology. Clin Microbiol Infect.

[2] Dijkshoorn L, Nemec A, Seifert H (2007). An increasing threat in hospitals: multidrug-resistant *Acinetobacter baumannii*. Nat Rev Microbiol.

[3] Peleg AY, Seifert H, Paterson DL (2008). *Acinetobacter baumannii*: emergence of a successful pathogen. Clin Microbiol Rev.

[4] Poirel L, Menuteau O, Agoli N, Cattoen C, Nordmann P (2003). Outbreak of extended-spectrum β-lactamase VEB-1-producing isolates of *Acinetobacter baumannii* in a French Hospital. J Clin Microbiol.

[5] Lockhart SR, Abramson MA, Beekmann SE, Gallagher G, Riedel S, Diekema DJ (2007). Antimicrobial resistance among Gram-negative bacilli causing infections in intensive care unit patients in the United States between 1993 and 2004. J Clin Microbiol.

[6] Falagas ME, Karveli EA (2007). The changing global epidemiology of *Acinetobacter baumannii* infections: a development with major public health implications. Clin Microbiol Infect.

[7] Zarrilli R, Pournaras S, Giannouli M, Tsakris A (2013). Global evolution of multidrug-resistant *Acinetobacter baumannii* clonal lineages. Int J Antimicrob Agents.

[8] Sunenshine RH, Wright M-O, Maragakis LL, Harris AD, Song X, Hebden J (2007). Multidrug-resistant *Acinetobacter* infection mortality rate and length of hospitalization. Emerg Infect Dis.

[9] Metzker ML (2010). Sequencing technologies—the next generation. Nat Rev Genet.

[10] Kinzler KW, Bigner SH, Bigner DD, Trent JM, Law ML, O&amp SJ (1987). Identification of an amplified, highly expressed gene in a human glioma. Science..

[11] Bolger AM, Lohse M, Usadel B (2014). Trimmomatic: a flexible trimmer for Illumina sequence data. Bioinformatics..

[12] Bankevich A, Nurk S, Antipov D, Gurevich AA, Dvorkin M, Kulikov AS (2012). SPAdes: a new genome assembly algorithm and its applications to single-cell sequencing. J Comput Biol.

[13] Bosi E, Donati B, Galardini M, Brunetti S, Sagot M-F, Lió P (2015). MeDuSa: a multi-draft based scaffolder. Bioinformatics.

[14] Hyatt D, Chen G-L, LoCascio PF, Land ML, Larimer FW, Hauser LJ (2010). Prodigal: prokaryotic gene recognition and translation initiation site identification. BMC Bioinformatics.

[15] Tm L, Sr E (1997). tRNAscan-SE: a program for improved detection of transfer RNA genes in genomic sequence. Nucleic Acids Res.

[16] Lagesen K, Hallin P, Rødland EA, Stærfeldt H-H, Rognes T, Ussery DW (2007). RNAmmer: consistent and rapid annotation of ribosomal RNA genes. Nucleic Acids Res.

[17] Benson G (1999). Tandem repeats finder: a program to analyze DNA sequences. Nucleic Acids Res.

[18] Grissa I, Vergnaud G, Pourcel C (2007). CRISPRFinder: a web tool to identify clustered regularly interspaced short palindromic repeats. Nucleic Acids Res.

[19] Altschul SF, Madden TL, Schäffer AA, Zhang J, Zhang Z, Miller W (1997). Gapped BLAST and PSI-BLAST: a new generation of protein database search programs. Nucleic Acids Res.

[20] Tatusov RL, Natale DA, Garkavtsev IV, Tatusova TA, Shankavaram UT, Rao BS (2001). The COG database: new developments in phylogenetic classification of proteins from complete genomes. Nucleic Acids Res.

[21] Marchler-Bauer A, Lu S, Anderson JB, Chitsaz F, Derbyshire MK, DeWeese-Scott C (2011). CDD: a conserved domain database for the functional annotation of proteins. Nucleic Acids Res.

[22] Wang H, Wang J, Yu P, Ge P, Jiang Y, Xu R (2017). Identification of antibiotic resistance genes in the multidrug-resistant *Acinetobacter baumannii* strain, MDR-SHH02, using whole-genome sequencing. Int J Mol Med.

[23] Kanehisa M, Goto S (2000). KEGG: Kyoto encyclopedia of genes and genomes. Nucleic Acids Res.

[24] Fedelstein J. PHYLIP (Phylogeny Inference Package). 1993.

[25] Gao F, Wang Y, Liu Y-J, Wu X-M, Lv X, Gan Y-R (2011). Genome sequence of *Acinetobacter baumannii* MDR-TJ. J Bacteriol.

[26] Fang Y, Quan J, Hua X, Feng Y, Li X, Wang J (2016). Complete genome sequence of *Acinetobacter baumannii* XH386 (ST208), a multi-drug resistant bacteria isolated from pediatric hospital in China. Genom Data..

[27] Cameron FH, Obbink JDO, Ackerman VP, Hall RM (1986). Nucleotide sequence of the AAD(2′) aminoglycoside adenylyltransferase determinant aadB. Evolutionary relationship of this region with those surrounding aadA in R538-1 and dhfrll in R388. Nucleic Acids Res..

[28] Zhou Y, Yu H, Guo Q, Xu X, Ye X, Wu S (2010). Distribution of 16S rRNA methylases among different species of Gram-negative bacilli with high-level resistance to aminoglycosides. Eur J Clin Microbiol Infect Dis.

[29] Hollingshead S, Vapnek D (1985). Nucleotide sequence analysis of a gene encoding a streptomycin/spectinomycin adenyltransferase. Plasmid.

[30] Wei Q, Jiang X, Yang Z, Chen N, Chen X, Li G (2009). dfrA27, a new integron-associated trimethoprim resistance gene from *Escherichia coli*. J Antimicrob Chemother.

[31] Bhullar K, Waglechner N, Pawlowski A, Koteva K, Banks ED, Johnston MD (2012). Antibiotic resistance is prevalent in an isolated cave microbiome. PLoS ONE.

[32] Bonnin RA, Nordmann P, Carattoli A, Poirel L (2013). Comparative Genomics of IncL/M-Type Plasmids: evolution by acquisition of resistance genes and insertion sequences. Antimicrob Agents Chemother.

[33] Bissonnette L, Champetier S, Buisson JP, Roy PH (1991). Characterization of the nonenzymatic chloramphenicol resistance (cmlA) gene of the In4 integron of Tn1696: similarity of the product to transmembrane transport proteins. J Bacteriol.

[34] Sköld O (2001). Resistance to trimethoprim and sulfonamides. Vet Res.

[35] Arcangioli M-A, Leroy-Sétrin S, Martel J-L, Chaslus-Dancla E (1999). A new chloramphenicol and florfenicol resistance gene flanked by two integron structures in *Salmonella typhimurium* DT104. FEMS Microbiol Lett.

[36] Chowdhury G, Pazhani GP, Nair GB, Ghosh A, Ramamurthy T (2011). Transferable plasmid-mediated quinolone resistance in association with extended-spectrum β-lactamases and fluoroquinolone-acetylating aminoglycoside-6′-*N*-acetyltransferase in clinical isolates of *Vibrio fluvialis*. Int J Antimicrob Agents.

[37] Agersø Y, Guardabassi L (2005). Identification of Tet 39, a novel class of tetracycline resistance determinant in *Acinetobacter* spp. of environmental and clinical origin. J Antimicrob Chemother.

[38] Greer ND (2006). Tigecycline (Tygacil): the first in the glycylcycline class of antibiotics. Proc Bayl Univ Med Cent..

[39] Poirel L, Naas T, Guibert M, Chaibi EB, Labia R, Nordmann P (1999). Molecular and biochemical characterization of VEB-1, a novel class a extended-spectrum β-Lactamase encoded by an *Escherichia coli* integron gene. Antimicrob Agents Chemother.

[40] Zong Z, Lü X, Valenzuela JK, Partridge SR, Iredell J (2008). An outbreak of carbapenem-resistant *Acinetobacter baumannii* producing OXA-23 carbapenemase in western China. Int J Antimicrob Agents.

[41] Paetzel M, Danel F, de Castro L, Mosimann SC, Page MGP, Strynadka NCJ (2000). Crystal structure of the class D β-lactamase OXA-10. Nat Struct Mol Biol.

[42] Poirel L, Marqué S, Héritier C, Segonds C, Chabanon G, Nordmann P (2005). OXA-58, a novel class D β-lactamase involved in resistance to carbapenems in *Acinetobacter baumannii*. Antimicrob Agents Chemother.

[43] Brown S, Amyes SGB (2005). The sequences of seven class D β-lactamases isolated from carbapenem-resistant *Acinetobacter baumannii* from four continents. Clin Microbiol Infect.

[44] Yong D, Toleman MA, Giske CG, Cho HS, Sundman K, Lee K (2009). Characterization of a new metallo-β-lactamase gene, blaNDM-1, and a novel erythromycin esterase gene carried on a unique genetic structure in *klebsiella pneumoniae* sequence type 14 from India. Antimicrob Agents Chemother.

